# Simple Co-Precipitation of Iron Minerals for the Removal of Phenylarsonic Acid: Insights into the Adsorption Performance and Mechanism

**DOI:** 10.3390/molecules28083448

**Published:** 2023-04-13

**Authors:** Lili Wang, Changchao Hu, Ze Yang, Songding Guo, Tingting Zhang, Shangyi Li

**Affiliations:** 1 Petroleum Exploration and Production Research Institute, SINOPEC, Beijing 100083, China; wangll.syky@sinopec.com; 2 Department of Environmental Science and Engineering, Research Centre for Resource and Environment, Beijing University of Chemical Technology, Beijing 100029, China; yzzb_0118@163.com (Z.Y.); guosongding123@163.com (S.G.); zhangtt@mail.buct.edu.cn (T.Z.)

**Keywords:** iron minerals, adsorption, phenylarsonic acid, ligand exchange interaction

## Abstract

In this study, three kinds of iron minerals, ferrihydrite, hematite, and goethite, were prepared by a simple coprecipitation method for the adsorption and removal of phenylarsonic acid (PAA). The adsorption of PAA was explored, and the influences of ambient temperature, pH, and co-existing anions on adsorption were evaluated. The experimental results show that rapid adsorption of PAA occurs within 180 min in the presence of iron minerals, and the adsorption process conforms to a pseudo-second-order kinetic model. The isothermal adsorption of PAA by ferrihydrite, goethite, and hematite agrees with the Redlich–Peterson model. The maximum adsorption capacities of PAA are 63.44 mg/g, 19.03 mg/g, and 26.27 mg/g for ferrihydrite, goethite, and hematite, respectively. Environmental factor experiments illustrated that an alkaline environment will significantly inhibit the adsorption of PAA by iron minerals. CO_3_^2−^, SiO_3_^2−^, and PO_4_^3−^ in the environment will also significantly reduce the adsorption performance of the three iron minerals. The adsorption mechanism was analyzed by FTIR and XPS, which indicated that ligand exchange between the surface hydroxyl group and the arsine group leads to the formation of an Fe-O-As bond, and electrostatic attraction between the iron minerals and PAA played an important role in the adsorption.

## 1. Introduction

Arsenic (As) is ubiquitous and humans are certainly exposed to arsenic via atmospheric air, groundwater, and food sources [1,2]. Most environmental arsenic contamination originates from anthropogenic activities which leads to millions of people experiencing life-threatening complications by drinking water poisoned and food produced from arsenic-contaminated soils irrigated with arsenic-contaminated water. Arsenic contamination has been noted as both a twentieth and twenty-first century catastrophe by researchers and authorities [3,4]. Unlike inorganic arsenic, the toxicity of organic arsenic compounds depends on the associated organic functional groups [5]. Chronic exposure of humans to organic arsenic contamination can lead to central nervous system disorders and optic nerve atrophy [6]. Although benzene organic arsenic compounds are generally considered less toxic than inorganic arsenic [7,8,9], organic arsenic substances may still be converted to inorganic arsenic through biotic and abiotic reactions in the natural environment, thereby increasing the risks of inorganic arsenic contamination [10]. However, Kroening et al., after biological toxicity research on the degradation products of arsenic-containing agents, found that the toxicity sequence, from high to low, was phenylarsine oxide, triphenylarsonic acid, phenylarsonic acid, diphenylarsonic acid, and pentavalent inorganic arsenic [11]. Therefore, the removal of organic arsenic from the environment needs to be closely monitored.

The removal of arsenic from water bodies can be performed by chemical flocculation, membrane filtration, and adsorption [12,13,14,15,16] to separate arsenic from the contaminated media. Due to the advantages of simple operation, high removal efficiency, low economic cost, and renewable solid and sustainable utilization, adsorption technologies for removing arsenic from sewage stand out among many treatment technologies. The adsorption performance of the adsorbent materials is the key to removing arsenic from water [17]. Compared with other functional materials, such as activated carbon, clay minerals, and biomass materials, the adsorption and removal performance of iron-based materials for arsenic is better [18] and more cost effective. In studies of arsenic adsorption, iron minerals such as ferrihydrite, hematite (α-Fe_2_O_3_), and goethite (α-FeOOH) are the most widely used. Different iron minerals exhibit different adsorption properties due to the differences in their chemical structures. Ferrihydrite is the largest of the iron minerals in terms of specific surface area, which is up to 700 m^2^/g, followed by goethite at 8~200 m^2^/g, and hematite, which exhibits the smallest surface area at only 2~115 m^2^/g [19]. Xie et al. [20] completed experiments on iron mineral adsorption of arsenic acid and loxarsine to eliminate the difference in specific surface area between different materials, and the adsorption capacity was normalized and found to satisfy the trend ferrihydrite > hematite > goethite.

The behavior and mechanisms occurring at the interface between iron oxides and contaminant water are important for the study of typical contaminant migration and transformation. Donald Sparks [21,22,23] worked on the interactions between minerals and contaminants, including outer sphere coordination, inner sphere coordination, particle diffusion, cluster formation or deposition, film or surface diffusion, co-precipitation or doping, and desorption or dissolution processes. Similar studies on the surface behavior of iron oxides and pollutants are also available. Based on this, we studied the interfacial reaction between iron minerals and organic arsenic to gain further insight into the immobilization of organic arsenic by adsorption at the molecular or atomic level. Iron minerals mainly adsorb arsenic by forming inner sphere complexes and outer sphere complexes with arsenic contaminants, where inner sphere complexes are chemisorbed and outer sphere complexes are physically adsorbed; thus, the stability of inner sphere complexes is significantly higher than that of outer sphere complexes [24]. Further studies on the structure of inner sphere complexes have revealed that there are three types of inner sphere complexes, namely bidentate binuclear, bidentate mononuclear, and monodentate mononuclear, among which the bidentate binuclear structure is the most stable [25]. Ferrihydrite is a type of adsorbent material with a strong affinity for arsenic contaminants. Li et al. [26] found that 2-line ferrihydrite with a mesoporous structure possesses excellent adsorption characteristics for arsenic (As(III)) contaminants. Experimental results showed that As (III) contamination can be effectively removed within 2 h by this 2-line ferrihydrite, which is attributed to its special mesoporous structure and large surface area (133 m^2^·g^−1^). The As (III) loading capacity of this mesoporous 2-line ferrihydrite was up to 128 mg (As) g^−1^(Fe). Masato et al. [27] used XAFS to characterize ferrihydrite adsorbed with organic arsenic and found that PAA and diphenylarsonic acid formed bidentate binuclear and monodentate mononuclear inner sphere complexation models with ferrihydrite, respectively. Goethite, a highly crystalline and stable iron mineral, is dehydroxylated to hematite when the temperature exceeds 120 °C. Compared with goethite, hematite has the same high crystallinity but a slightly weaker thermal stability. Goethite and hematite are also widely used for the removal of various arsenic contaminants due to their abundance, easy preparation, and low cost.

There is no doubt that different crystalline surfaces of iron minerals have different adsorption effects on arsenic contaminants, and the reactive sites on the crystalline surfaces are the key factors controlling the adsorption process. Yan et al. [28] concluded that arsenite and arsenate formed single dentate complexes with (001) crystals and double-dentate complexes with (110) and (214) crystals, determined by XAFS characterization and density functional theory calculations. Chen et al. [29] indicated that DFT-calculated adsorption energies and charge density redistributions for various surface complexes on different charged (101) and (210) facets are consistent with the trends observed in batch measurements, suggesting that the observed behavior reflects the primary controlling influence of goethite surface chemistry at the molecular scale. Cao et al. [30] found that organic arsenic adsorbed on two facets (e.g., (001) and (012)) of hematite through the same non-protonated inner sphere coordination but in different molecular configurations. Surface complexation models were set up to analyze the preferred coordination geometries dependent on the facets, and the (012) facet was found to favor PAA adsorption more than the (001) facet.

In this work, three common iron minerals, including ferrihydrite, goethite, and hematite, were prepared by the co-precipitation method as adsorbent materials for the adsorption and removal of PAA. The physicochemical properties of the three iron minerals were characterized by scanning electron microscopy, N_2_ adsorption and desorption tests, Fourier infrared spectroscopy, and X-ray energy spectroscopy. The differences in the adsorption capacity of the three iron minerals for PAA were evaluated by isothermal adsorption, adsorption thermodynamics, and adsorption kinetics experiments. The effects of environmental factors such as pH and coexisting ions on the adsorption performance of the iron minerals were investigated, and the possible adsorption mechanisms were inferred by material characterization before and after adsorption.

## 2. Results

### 2.1. Characterizations of Ferrihydrite, Hematite, and Goethite

**Figure 1:**
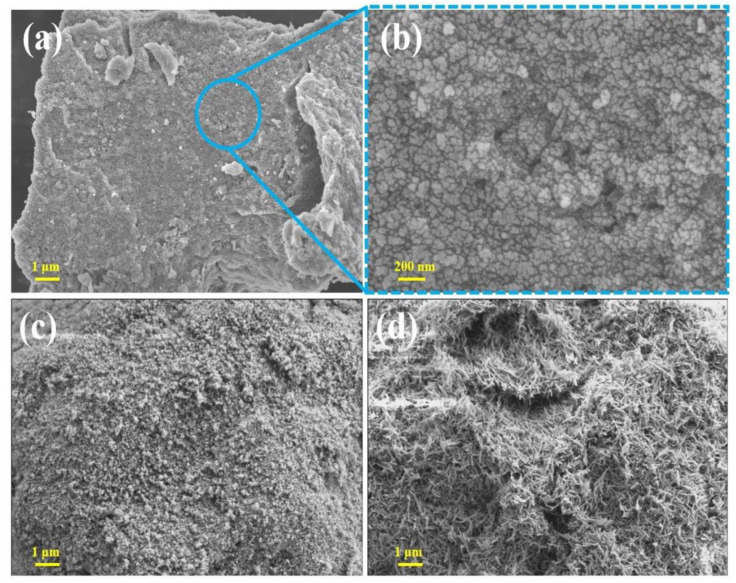
SEM images of (**a**) ferrihydrite, (**b**) ferrihydrite with a higher magnification, (**c**) hematite, and (**d**) goethite.

**Figure 2:**
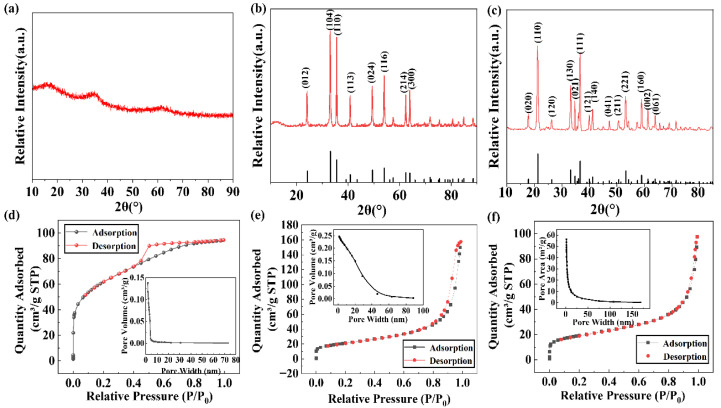
XRD patterns of iron minerals with different crystallinity: (**a**) ferrihydrite, (**b**) hematite, and (**c**) goethite. N_2_ adsorption/desorption isotherms and pore size distribution of (**d**) ferrihydrite, (**e**) hematite, and (**f**) goethite. a.u.—arbitrary units.

Figure 1 shows the SEM image of ferrihydrite (a), hematite (b), and goethite (c). The ferrihydrite exhibited an irregular particle structure with a rough surface and abundant pores [31]. Fine particles were agglomerated on the surface of hematite, which was very rough. Goethite showed a typical 3D needle-like structure, which had a certain mechanical stability against wear. The XRD characterization results are shown in Figure 2a–c. The XRD pattern of ferrihydrite shows wide diffraction peaks at 36° and 62°, indicating that the preparation is definitely an amorphous iron mineral. In contrast, goethite and hematite exhibited multiple diffraction peaks in the range of 10°~80°, and the peaks are narrow and pointed, indicating that synthetic goethite and synthetic hematite showed high crystallinity [32]. By comparing with the XRD patterns of the standard goethite and hematite, it was found that the two kinds of iron minerals are in good conformity with the standard materials. During the synthesis of iron minerals, it was found that the pH of the solution and the synthesis temperature played an important role in their crystal transformation.

The pore structure analysis of the three iron minerals is shown in Figure 2d–e. The adsorption isotherm of ferrihydrite is more in line with a type I adsorption isotherm, which may theoretically provide space for physical adsorption via pore filling. Both goethite and hematite belong to the type IV adsorption isotherm with H1 hysteresis loops, indicating that the pore size of the adsorbent material distribution was more uniform. The specific surface areas of ferrihydrite, goethite, and hematite were 214 m^2^/g, 65 m^2^/g, and 73 m^2^/g, respectively. The pore volumes were 0.146 cm^3^/g, 0.142 cm^3^/g, and 0.246 cm^3^/g, respectively. Additionally, the tested pore diameters were 2.719 nm, 8.502 nm, and 13.271 nm, respectively.

### 2.2. PAA Adsorption Performance of Ferrihydrite, Hematite, and Goethite

**Figure 3:**
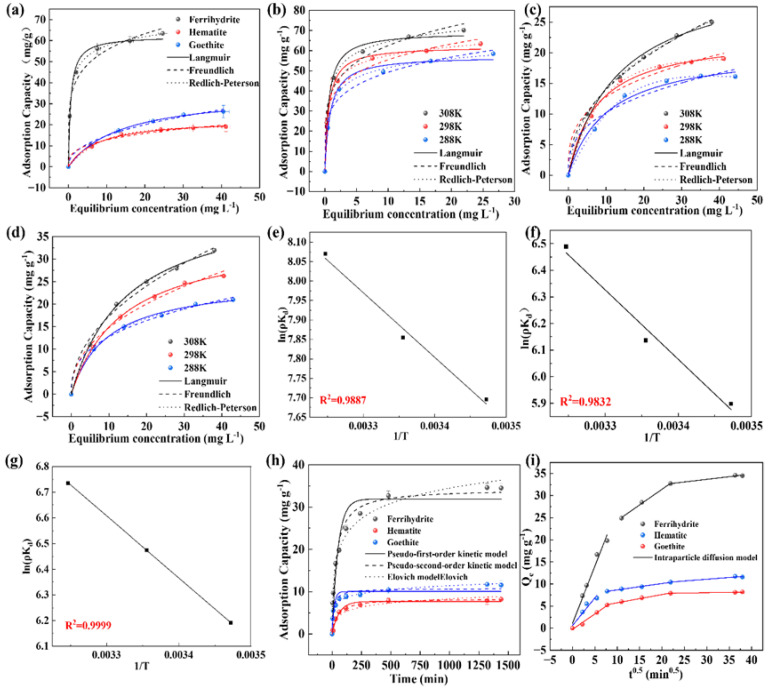
(**a**) Isothermal adsorption model of iron minerals, isothermal adsorption models of (**b**) ferrihydrite, (**c**) goethite, and (**d**) hematite at different temperatures. Van ‘t Hoff curve of adsorption of PAA by (**e**) ferrihydrite, (**f**) goethite, and (**g**) hematite. (**h**) Kinetic model of iron minerals. (**i**) Intragranular diffusion model.

Freundlich, Langmuir, and Redlich–Peterson isotherms were used to fit the experimental data in Figure 3a. The specific fitting parameters are shown in Table 1. The Langmuir isothermal adsorption model is used to describe a monolayer adsorption process, assuming that the active sites on the adsorbent surface are uniformly distributed. The Freundlich isothermal adsorption model is an empirical model, which can describe multilayer adsorption on the heterogeneous surface of an adsorbent [33]. The Redlich–Peterson isothermal adsorption model is a three-parameter model combining the features of Langmuir and Freundlich isothermal adsorption models for describing the adsorption process in homogeneous and non-homogeneous systems, while also considering the interaction between the adsorbent active site and the adsorbed material. The results showed that in contrast with the results fitted by the Langmuir or Freundlich models, the Redlich–Peterson isothermal model can achieve a higher R^2^ value (0.999) and it was suitable for describing the adsorption process of PAA in iron minerals. In the fitting parameter, β deviates from 1, indicating that the adsorption process is mainly controlled by chemisorption [34]. According to the fitting results, the maximum adsorption capacities of ferrihydrite, goethite, and hematite are 62.21 mg/g, 22.99 mg/g, and 35.58 mg/g, respectively.

In the adsorption process, the temperature is an important factor affecting the adsorption effect. At three different reaction temperatures of 288K, 298K, and 308K, ferrihydrite, goethite, and hematite were used to absorb PAA to explore the effect of temperature on the adsorption process. The experimental results are shown in Figure 3b–d. The adsorption capacity of three iron minerals for PAA increases with the increase in adsorption temperature, and the isothermal adsorption models are consistent with the Redlich–Peterson model, which agrees with the results of the isothermal adsorption experiments. The experimental data were fitted and analyzed using the Van ‘t Hoff equation, and the feasibility of adsorption was determined by analyzing Δ*G*, Δ*H*, and Δ*S* (Figure 3e–g). The fitting results of thermodynamic experimental data are shown in Tables S1 and S2. Under different temperature conditions, the Δ*G* in the adsorption process of PAA for all three iron minerals is all less than 0, and Δ*G* decreases with the increase in temperature [35], indicating that the degree of thermodynamic responsivity increases with the increase in temperature. A Δ*S* > 0 indicates that the adsorption process of the three iron minerals on PAA is a spontaneous entropy increasing process. A Δ*H* > 0 indicates that the adsorption process is a endothermic process and a high temperature is favorable for the adsorption reaction [36].

The experimental results of adsorption kinetics are shown in Figure 3h. In the case of the three iron minerals, the adsorption capacity of PAA increases rapidly within 3 h after the adsorption begins, and then the adsorption rate slows down gradually and finally reaches the adsorption equilibrium. The adsorption equilibrium of goethite and hematite was reached very quickly, while the adsorption equilibrium of ferrihydrite was slightly delayed in contrast with the former materials. To further accurately describe the adsorption behavior of iron minerals in PAA, pseudo-first-order kinetic, pseudo-second-order kinetic, and Elovich models were fitted to the adsorption results. Studies have shown that the pseudo-first-order kinetic model tends to describe the initial concentration of pollutants with a relatively high number of active sites on the adsorbent, which is usually used to describe the physical adsorption process [37], while the pseudo-second-order kinetic model is more suitable to describe the initial concentration of pollutants with a relatively low number of active sites on the adsorbent, which is usually used to describe the chemisorption process. It is easy to see from Table 2 that the fitting results of the pseudo-second-order kinetic model for the adsorption of PAA by ferrihydrite, goethite, and hematite (R^2^: 0.98, 0.99, and 0.96, respectively) are better than those of the pseudo-first-order kinetic model (R^2^: 0.93, 0.98, and 0.89, respectively), indicating that chemisorption is likely to occur in the adsorption of PAA by the three iron minerals [38]. Meanwhile the fitted results of the adsorption rates of ferrihydrite, goethite, and hematite were 0.00085 mg·g^−1^·min^−1^, 0.00267 mg·g^−1^·min^−1^, and 0.00725 mg·g^−1^·min^−1^, respectively, indicating that hematite had the fastest adsorption rate of PAA, followed by goethite, and ferrihydrite had the slowest adsorption rate. The fitting results of the Elovich model also verified that chemisorption occurred between the iron minerals and PAA.

To explore whether the velocity control step of the adsorption process is external diffusion or internal diffusion, the intraparticle diffusion model was chosen to fit the kinetic data again. The fitting results of each adsorbent in Figure 3i are divided into three linear parts, which proves the complexity of the adsorption process and also indicates that the adsorption process includes three diffusion steps [39]: (1) the external diffusion process of PAA from the liquid PAAe to the surface of iron minerals; (2) the internal diffusion process of PAA from the adsorbent surface into the pores after adsorption to the internal active site; and (3) the process of adsorption gradually reaching equilibrium. As shown in Table 3, it can be observed that the adsorption rate of ferrihydrite is much larger than that of goethite in stage I. As the adsorption process continues, the adsorption rate of ferrihydrite in stages II and III has decreased to a very low level, indicating that ferrihydrite quickly reaches saturation due to rapid adsorption, leading to a decrease in the mass transfer driving force. According to the theory of Weber and Morris, intraparticle diffusion can be considered the rate-controlling step when the solute adsorption, *Q*_e_, versus t^0.5^ is a straight line through the origin [40]. According to the fitting results in Table 3, it was found that the fitting of the adsorption results for all iron minerals had a strong linear relationship. It is noteworthy that none of them passed through the origin, proving that the adsorption process was influenced by other mechanisms besides the intraparticle diffusion process. It is inferred that a thick boundary layer may be present on the surface of ferrihydrite, where PAA needs to approach the surface of the adsorbent from the main body of the liquid PAA by membrane diffusion and then enter the adsorbent by intraparticle diffusion internally.

### 2.3. Effect of Main Parameters on the Adsorption of PAA

**Figure 4:**
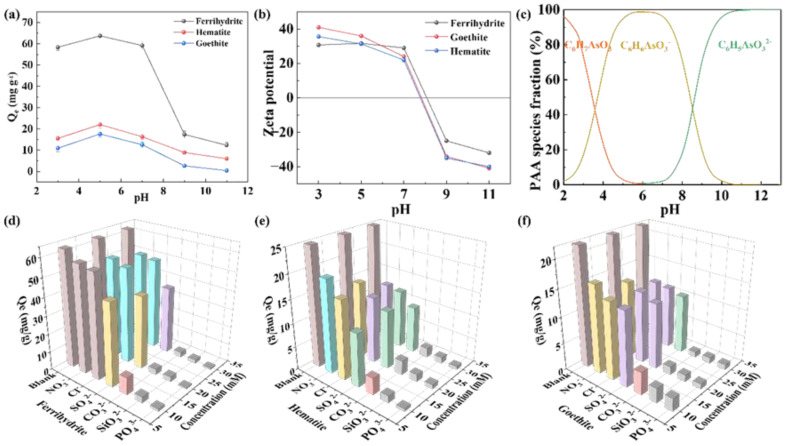
(**a**) The effect of different pH conditions on the adsorption effect of iron minerals on PAA. (**b**) Zeta potential diagram of iron minerals. (**c**) Relationship between the presence of PAA and pH. Effect of coexisting anions on adsorption of PAA by (**d**) ferrihydrite, (**e**) hematite, and (**f**) goethite.

The adsorption effects of iron minerals under different pH conditions were investigated. As can be seen from Figure 4a, all iron minerals exhibit remarkable adsorption properties, and the adsorption properties change little in the range of pH = 3–7. However, in the range of pH = 7–11, the adsorption capacity decreases sharply with the increase in pH. Under acidic conditions, there is little difference in the adsorption performance of ferrihydrite. When pH = 5, the adsorption capacity of ferrihydrite reaches the maximum, at 65 mg/g. Similarly, hematite and goethite also obtained the maximum adsorption capacity at pH = 5, which were 22 mg/g and 17 mg/g, respectively. The effect of pH on the adsorption properties is usually closely related to the electrostatic attraction between adsorbed materials and pollutants. Hence, we measured the zeta potential of three iron minerals to analyze the change in surface charge. From Figure 4b, we can see that the isoelectric points of ferrihydrite, hematite, and goethite are very close to each other at pH = 8. In this case, when the environmental pH is less than 8, the surface of the three iron minerals is positively charged [41], and when the environmental pH is greater than 8, the surface of the three iron minerals is negatively charged [42]. In addition, the pH affects the dissociation equilibrium and morphological changes of PAA molecules in an aqueous solution. Visual MINTEQ was used to simulate the dissociation of PAA in water. According to Figure 4c, it is not difficult to see that PAA is present in molecular form at pHs less than 6. In the range of pH = 2–11, C_6_H_6_AsO_3_^−^ is generated due to the loss of one proton, and in the range of pH = 5–8, the content of C_6_H_6_AsO_3_^−^ would be close to 100% [43]. C_6_H_5_AsO_3_^2−^ is formed by the loss of two protons when the pH greater is than 6, which may mean that when the pH is higher than the isoelectric point of ferrihydrite, deprotonation will occur on its surface and it will have a negative charge. Meanwhile, according to the morphological distribution of PAA under alkaline conditions, PAA mainly exists in the forms of C_6_H_6_AsO_3_^−^ and C_6_H_5_AsO_3_^2−^, and the electrostatic repulsion between them will prevent a certain amount of PAA from being adsorbed to ferrihydrite, thus reducing the adsorption capacity. Under the conditions of strong acidity, ferrihydrite has a positive charge due to protonation; the adsorption capacity of PAA can be improved theoretically through electrostatic attraction. However, under these conditions, PAA exists in the form of molecules, and the electrostatic attraction between them will be relatively weakened, resulting in a decrease in the adsorption capacity.

The effect of coexisting ions in water on the adsorption of PAA by iron minerals was further considered. The adsorption of PAA by iron minerals in the presence of NO_3_^−^, Cl^−^, SO_4_^2−^, CO_3_^2−^, SiO_3_^2−^, and PO_4_^3−^ was investigated in Figure 4d–f. The adsorption effect of the three iron minerals on PAA decreases with the increase in anion concentration. From observing the data in ferrihydrite, we can see that the removal of PAA decreased by 8.3% in the presence of 20 mM NO_3_^−^ and 10.8% in the presence of 20 mM Cl^−^. In hematite, the removal of PAA decreased by 8.9% in the presence of 20 mM NO_3_^-^ and 11% in the presence of 20 mM Cl^−^. The adsorption of PAA by goethite decreased by 7.7% in the presence of 20 mM NO_3_^−^ and 8.6% in the presence of 20 mM Cl^−^. CO_3_^2−^, SiO_3_^2−^, and PO_4_^3−^ all have strong inhibitory effects on the three iron minerals, as their adsorption capacity almost decreased to 0 at the concentration of 10 mM. On the one hand, CO_3_^2−^ hydrolyzes in water to produce OH^−^, which increases the environmental pH value and is not conducive to the adsorption of PAA by iron minerals. On the other hand, SiO_3_^2−^ and PO_4_^3−^ both have a similar tetrahedral spatial structure with AsO_4_^3−^, and also form endosphere complexes with hydroxyl groups on the surface of iron minerals, which leads to the adsorption inhibition of PAA [41]. The experimental results also show that SO_4_^2−^ can inhibit the adsorption of phenylarsonic acid on iron minerals to a certain extent. Xie et al., obtained similar results when exploring the performance of metal oxides in adsorbing phenylarsonic acid compounds, which may be due to the SO_4_^2−^ competing with PAA for the active site on the iron minerals, thus affecting the iron mineral’s adsorption of PAA [20,44]. Figure S1 presents the effect of humic acid on the adsorption process of PAA with ferrihydrite, with the results showing that the adsorption capacity was virtually unchanged. Namely, in the system containing organic matter, ferrihydrite still exhibited a good adsorption performance. Figure S2 presents the material reusability in the adsorption process of PAA by ferrihydrite, with the results showing that the adsorption capacity of ferrihydrite decreased by around 40% after two cycles.

As an in situ solution to pollution problems, a permeable reactive wall (PRB) could remove pollutants at their original position during groundwater remediation. Therefore, a column filled with ferrihydrite was used to simulate the PRB process for PAA adsorption under different flow rates (Figure S3). The value of C/C_0_ was 0.1 and 0.95 represented the penetration and depletion point, respectively. It can be seen from the figure that when the flow rates were 5 mL/min and 10 mL/min, the fixed bed of ferrihydrite was penetrated after only 5 pore volumes, and the depletion points appeared via 410 pore volumes and 240 pore volumes, respectively. When the flow rate was 1 mL/min, the fixed bed was penetrated through 35 pore volumes and reached the exhaustion point through 465 pore volumes. Therefore, the slower flow rate (1 mL/min) better facilitated the removal of phenylarsonic acid by an adsorption fixed bed owing to delayed the penetration and depletion point.

### 2.4. Mechanism of Adsorption

**Figure 5:**
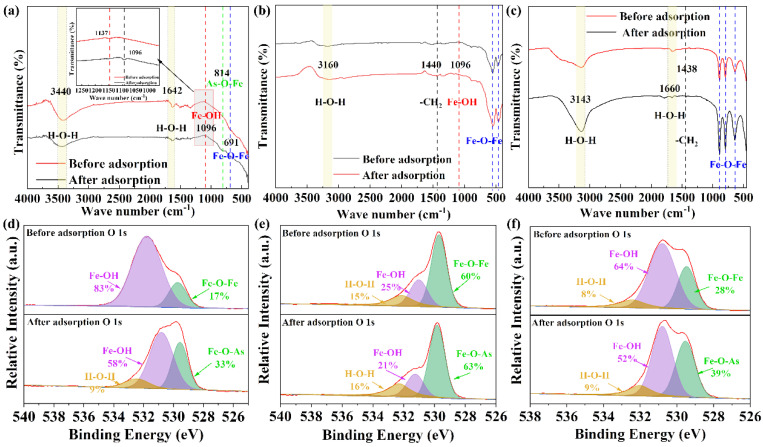
FTIR spectra of (**a**) ferrihydrite, (**b**) hematite, and (**c**) goethite. O 1s XPS spectra of (**d**) ferrihydrite, (**e**) hematite, and (**f**) goethite.

Figure 5a–c shows the FTIR spectra of the three different iron minerals. According to Figure 5a, after the adsorption of PAA by ferrihydrite, relatively strong peaks appeared near 3440 cm^−1^ and 1642 cm^−1^, which was attributed to the stretching vibration of hydroxyl groups in water [45]. The peaks at 691 cm^−1^ and 419 cm^−1^ belong to the vibration of the Fe-O bond. Before adsorption, the stretching vibration of hydroxyl groups in hydroxyl oxide appeared at 1137 cm^−1^. After adsorption, the characteristic peak was redshifted to 1096 cm^−1^. This phenomenon may be caused by hydrogen bonding between the hydroxyl group in the ferrihydrite and PAA, which decreases the bonding force constant of the hydroxyl group. At the same time, the characteristic peak of As-O-Fe was formed at 814 cm^−1^ in the adsorbed ferrihydrite{10}, which further proved that the adsorption of PAA on ferrihydrite occurred. It can be seen from Figure 5b that there were some obvious characteristic peaks in the FTIR spectra of hematite. Before and after adsorption, the stretching vibration peaks of the hydroxyl groups in water appeared near 3160 cm^−1^ and 1671 cm^−1^ in the two samples. The peaks at 556 cm^−1^ and 474 cm^−1^ belong to the vibration of the Fe-O bond [46]. Before adsorption, the stretching vibration belonging to hydroxyl group appeared at 1136 cm^−1^ for hematite. After adsorption, the characteristic peak was redshifted to 1094 cm^−1^. The reason for this phenomenon may be that the hydroxyl group in hematite is hydrogen-bonded with PAA, which reduces the bonding force constant of the hydroxyl group. At the same time, the peaks of the Fe-O bond moved from 556 cm^−1^ and 474 cm^−1^ to 550 cm^−1^ and 461 cm^−1^, respectively. However, the characteristic peak of As-O-Fe still did not appear in the spectrum of hematite after adsorption [20]. In addition, compared with before adsorption, after adsorption, hematite has an obvious peak at 1440 cm^−1^, which represents the characteristic vibration of -CH_2_ [47]. Compared with the FTIR spectrum of ferrihydrite, some obvious characteristic peaks appeared in the FTIR spectra of goethite (Figure 5c). Before and after adsorption, the two samples showed strong peaks near 3143 cm^−1^ and 1660 cm^−1^, which were caused by the stretching vibration of hydroxyl groups in water. Strong peaks of Fe-O bonds appeared at 892 cm^−1^, 795 cm^−1^, and 639 cm^−1^. Unfortunately, the characteristic peak of Fe-O-As did not appear in the spectra after adsorption, but it did not mean that there was no chemical interaction between goethite and PAA, but was probably due to the low adsorption capacity [48]. In addition, compared with before adsorption, goethite has an obvious peak at 1438 cm^−1^ after adsorption, which was attributed to the characteristic vibration of -CH_2_ [47].

To further explore the PAA adsorption mechanism of the three iron minerals, the XPS analysis results are shown in Figure 5d–e. A comparison of the O 1s orbital XPS spectra of the iron minerals found three peaks at 532, 529, and 531 eV, which correspond to water, Fe-O-Fe, and Fe-OH, respectively [43]. It can be seen from Figure 5d that ferrihydrite has only two oxygen-containing functional groups before adsorption, namely Fe-OH and Fe-O-Fe, with contents of 83% and 17%, respectively. This also shows that ferrihydrite contains a large number of amorphous iron oxide functional groups on its surface, which gives ferrihydrite its excellent adsorption capacity. After adsorption, three absorption peaks appeared in ferrihydrite, showing that ferrihydrite adsorbed about 10% of the water molecules in the adsorption process of PAA, the content of Fe-OH decreased by 25%, and the content of Fe-O-Fe increased by 16%. It can be seen from Figure 5e that before the adsorption by hematite, the percentages of H-O-H, Fe-OH, and Fe-O-Fe were 15%, 25%, and 60%, respectively, while the percentage of Fe-OH was relatively small and the content of Fe-O-Fe was large, which proves the dehydroxylation of iron oxides after the high-temperature synthesis. The percentage of water molecules in hematite after adsorption was 16%, almost unchanged, while the percentage of hydroxyl groups decreased to 31% and the content of Fe-O-As increased to 63%. Figure 5f shows that before the adsorption of goethite, the percentages of H-O-H, Fe-OH, and Fe-O-Fe were 8%, 64%, and 28%, respectively. After adsorption, the percentage of H-O-H in goethite is 9%, which was almost unchanged, and the percentage of Fe-OH decreased to 52%, while the percentage of Fe-O-As was 39%. Furthermore, Figures S4–S6 show the As XPS spectra of the used iron minerals, indicating obvious PAA adsorption. The overall adsorption mechanism diagram is shown in Figure S7.

## 3. Materials and Methods

### 3.1. Chemicals

All chemicals were analytically pure and used without further purification. PAA (C_6_H_7_AsO_3_) was purchased from the Tokyo Chemical Industry. Ferric chloride hexahydrate (FeCl_3_⋅6H_2_O), ferric nitrate nonahydrate (Fe(NO_3_)_3_⋅9H_2_O), sodium chloride (NaCl), sodium carbonate (Na_2_CO_3_), sodium sulphate (Na_2_SO_4_), sodium silicate (Na_2_SiO_3_), and sodium phosphate (Na_3_PO_4_) were purchased from the Macklin Biochemical Co. Hydrochloric acid (HCl, 36%) and sodium hydroxide (NaOH) were purchased from the Beijing Chemical Factory Co.

### 3.2. Preparation of Ferrihydrite, Goethite, and Hematite

For ferrihydrite, a certain amount of ferric chloride hexahydrate was dissolved in deionized water to prepare a ferric chloride solution with a concentration of 0.01 mol/L, then it was sonicated for 20 min so that ferric chloride was evenly dispersed. The beaker containing the ferric chloride solution was placed in a mixer with a rotation speed set to 600 r/min. The pH value was adjusted by a sodium hydroxide solution with a concentration of 5 mol/L until the pH was 7. After stirring for 30 min, the solution was aged for 12 h. After washing with deionized water 5 times, centrifugal separation was carried out, and the obtained iron mud was freeze-dried. In the pre-experimental stage, we synthesized ferrihydrite by coprecipitation at room temperature. When the synthesis temperature was raised to 70 ℃, the generated iron minerals were still ferrihydrite, and the adsorption effect of PAA was very similar due to the unchanged crystal type. When the reaction pH was adjusted to 12 at the reaction temperature of 70 ℃, the iron minerals changed from red-brown to the characteristic earthy yellow of goethite. Similarly, in the process of preparing hematite, we raised the synthesis temperature to 90 ℃ and controlled the pH of the reaction to obtain hematite. Cudennec et al. studied the conversion conditions of ferrihydrite to goethite and hematite and came to a similar conclusion; high temperature and neutral pH are more conducive to the formation of hematite, while low pH (pH = 2–5) and high pH (pH = 10–14) are more conducive to the formation of goethite.

For goethite, a certain amount of ferric nitrate nonahydrate was dissolved in deionized water to prepare a ferric nitrate solution at a concentration of 0.01 mol/L. The beaker containing the ferric nitrate solution was placed into a water bath and 5 mol/L sodium hydroxide solution was added to the solution until the pH was 12, and it was left to stand for 1 h. Then, the temperature of the water bath was set at 70 ℃ for aging for 12 h. After the reaction temperature dropped to room temperature, the reaction products were washed with deionized water to neutralize them, and the iron mud was separated by centrifugation and freeze-dried.

For hematite, a 200 mL ferric nitrate solution with a concentration of 0.2 mol/L was prepared in a beaker. Then, 24 mL of 5 mol/L sodium hydroxide solution and 20 mL of 1 mol/L potassium bicarbonate solution was added into the ferric nitrate solution at the same time. The mixture was placed in a water bath at 90 ℃ and heated to age for 12 h. The thermally treated sample was then cooled to room temperature by air and washed with deionized water 3 times. Finally, the sample was centrifuged to separate the iron mud and then freeze-dried.

### 3.3. Adsorption Experiments

#### 3.3.1. Isothermal Adsorption Experiment

PAA solutions (50 mL) with concentrations of 10, 20, 30, 40, and 50 mg/L were placed several conical bottles, and 0.02 g of adsorption material was added. The conical mouth of the bottle was sealed and placed in a 150 rpm, 298 K constant temperature air shaker for oscillation. When adsorption equilibrium was reached after 1440 min, a needle tube was used for sampling. The collected liquid was treated through a 0.22 μm filter. The equations for isothermal adsorption are shown as follows (Equations (1)–(3)):

Langmuir isotherm:

(1) $$Q_{e}=\frac{Q_{\max}C_{e}K_{L}}{1+C_{e}K_{L}}$$

Freundlich isotherm:

(2) $$Q_{e}=K_{f}C_{e}{}^{\frac{1}{n}}$$

Redlich–Peterson isotherm:

(3) $$Q_{e}=\frac{C_{e}K_{R}}{1+\alpha C_{e}{}^{\beta }}$$

where *Q*_max_ (mg·g^−1^) is the saturated monolayer adsorption capacity and *K*_L_ (L·mg^−1^) is the Langmuir constant related to the adsorbent energy. *K*_F_ (mg·g^−1^) and n are Freundlich constants, which are related to adsorption capacity and relative adsorption strength, respectively. *K*_R_ (mg·g^−1^), α, and β are constants of the Redlich–Peterson model.

The experimental process to determine the influence of different temperatures is as follows:

A certain amount of adsorbent was put into conical bottles with a concentration of 0.4 g·L^−1^, and 50 mL of solution was added to each conical flask with concentrations of 5 mg·L^−1^, 15 mg·L^−1^, 20 mg·L^−1^, 25 mg·L^−1^, and 55 mg·L^−1^. The conical mouth of the bottle was sealed and oscillated in a constant temperature air shaker at 150 rpm at temperatures of 288 K, 298 K, and 308 K. When adsorption equilibrium was reached after 1440 min, needle tube sampling was used. The collected liquid was treated through a 0.22 μm filter. The calculation of *K*_d_, Δ*G*, and ln(*ρK*_d_) was performed (Equations (4)–(6)):

(4) $$K_{d}=\frac{Q_{e}}{C_{e}}$$

(5) $$\Delta G=-\mathrm{RT}\cdot \ln\left(\rho K_{d}\right)$$

(6) $$\ln\left(\rho K_{d}\right)=\frac{\Delta S}{R}-\frac{\Delta H}{\mathrm{RT}}$$

where *K*_d_ is the allocation coefficient, which is the ratio of adsorption capacity (*Q*_e_, mg·g^−1^) of the material at equilibrium to pollutant concentration (*C*_e_, mg·L^−1^) at equilibrium adsorption. *ρ* is the density of water (g·L^−1^), R is the gas mole constant (R = 8.314 J·mol^−1^·K^−1^), T is the adsorption temperature (K), Δ*H* is the enthalpy change (kJ·mol^−1^), Δ*S* is the entropy change (J·mol^−1^·K^−1^), and Δ*G* is the Gibbs free energy (kJ·mol^−1^).

#### 3.3.2. Adsorption Kinetics Experiment

An amount of 50 mL of PAA solution with a certain concentration was placed into a conical bottle, 0.02 g of adsorption material was added, the conical bottle was sealed with tin foil, and the solution was oscillated in a constant temperature air shaker at 150 rpm and 298 K. Samples were taken with 1 mL syringes at 0 min, 10 min, 30 min, 60 min, 120 min, 240 min, 360 min, 720 min, 1320 min, and 1440 min after the reaction began, and the collected samples were stored in a refrigerator after being coated. Equations (7)–(10) exhibit the adsorption kinetics models:

Pseudo-first-order kinetic model:

(7) $$\ln\left(Q_{e}-Q_{t}\right)=\ln Q_{e}-k_{1}t$$

Pseudo-second-order kinetic model:

(8) $$\frac{t}{Q_{t}}=\frac{1}{k_{2}Q_{e}{}^{2}}+\frac{t}{Q_{e}}$$

Elovich model:

(9) $$Q_{t}=\frac{1}{\beta }\ln\left(1+\alpha \cdot \beta \cdot t\right)$$

Intragranular diffusion model:

(10) $$Q_{t}=k_{\mathrm{id}}t^{0.5}+C$$

where *Q*_e_ and *Q*_t_ are the adsorption capacities (mg·g^−1^) at adsorption equilibrium and at time *t* (min) after adsorption, respectively. *k*_1_ (min^−1^) and *k*_2_ (g·mg^−1^·min^−1^) are the adsorption rate constants of pseudo-first-order kinetics and pseudo-second-order kinetics, respectively. *α* (mmol·g^−1^·h^−1^) and *β* (g·mmol^−1^) represent the initial adsorption rate and desorption constant of the Elovich model. *k*_id_ (mg·g^−1^·min^−1/2^) is the rate constant of the intragranular diffusion model and C is the intercept, which can give information about the material boundary layer thickness. In general, the larger the intercept, the greater the contribution of surface adsorption to the rate.

#### 3.3.3. Influencing Factor Testing

The initial concentration of PAA was set to 50 mg·L^−1^, and six anions, including nitric acid, chloride ion, sulfate, carbonate, silicate, and phosphate, were added to the solution at dosages of 10, 20, and 30 mM. The concentration of the adsorbent was 0.4 g·L^−1^. The initial pH was adjusted to 3, 5, 7, 9, and 11 by using 0.1 M of HCl and 0.1 M of NaOH. All reactions were carried out in conical bottles and sealed with tin foil during adsorption. The solution was placed in a 150 rpm, 298 K constant temperature air shaker for 1440 min. After this time, a needle tube was used for sampling, and the collected liquid was passed through a 0.22 μm filter 1 mL at a time.

### 3.4. Characterization and Analytical Methods

SEM images of samples were taken using a scanning electron microscope (Gemini 300, ZEISS, Oberkochen, Germany). XRD was performed on the sample by an X-ray diffractometer (Ultima IV, Rigku, Japan) using Cu Kα radiation, λ = 0.15406 A. The scanning range was 10°–80° and the scanning speed was 10° min^−1^. FT-IR spectra of the samples were measured using a Fourier infrared spectrometer (Scientific Nicolet iS10, ThermoFischer, Waltham, MA, USA). The scanning wavenumber ranges from 4000 to 400 cm^−1^, with 32 scans, and the resolution was 4 cm^−1^. For the FTIR analyses after adsorption, we carried out filtration and freeze drying to obtain the measurement sample. The Zeta potential was analyzed using the nanometer particle size and a Zeta potential analyzer (Zetasizer Nano ZS90, Malvern, UK). The BET curve of the sample was measured by using a fully automated specific surface and porosity analyzer, BET (ASAP 2460 3.01, Micromeritics, USA). The degassing temperature was 423 K, the gas was N_2_, and the degassing time was 6 h. XPS was performed using an X-ray photoelectron spectrometer (ESCALAB 250Xi, ThermoFischer, USA). For the XPS analyses after adsorption, we carried out filtration and freeze drying to obtain the measurement sample. High-performance liquid chromatography (LC-6 Shimadzu, Elsichrom, Knivsta, Sweden) was used to detect the concentration of PAA with a C18 column (ZORBAX Agilent, Agilent Technologies, Santa Clara, MO, USA) at 298 K. The mobile PAA consisted of acetonitrile and 0.2% phosphoric acid solution. The mobile PAA ratio was 1:9 at the wavelength of 264 nm and the injection volume was 20 μL each time.

## 4. Conclusions

Ferrihydrite, hematite, and goethite were prepared by a simple coprecipitation method, and all exhibited good adsorption performance for PAA. The adsorption isotherm of ferrihydrite is more consistent with a type I isotherm, indicating that micropore filling is the main physical mechanism of ferrihydrite adsorption of PAA. According to the BET characterization results, the specific surface area of ferrihydrite is 214 m^2^/g, much higher than that of hematite (73 m^2^/g) and goethite (65 m^2^/g), and ferrihydrite has the best adsorption effect of PAA, at up to 63.44 mg/g. The adsorption kinetics experiment showed that the adsorption of PAA by the three iron minerals was more in line with a pseudo-second-order kinetic model, indicating that there was chemical adsorption between the three iron minerals and PAA molecules. To explore whether the speed control step of the adsorption process was external diffusion or internal diffusion, the intraparticle diffusion model was used to fit the kinetic data again. In addition to the internal diffusion process, the existence of a boundary layer on the surface of the iron minerals will undoubtedly increase the mass transfer resistance, thus affecting the adsorption process. Furthermore, the influence of the environmental pH and co-existing anions on the adsorption of PAA by iron minerals was considered. This study provides new motivation for the development of simple and efficient adsorption removal technologies for PAA. Finally, the adsorption mechanism was discussed through a characterization of FTIR and XPS spectra. The results showed that after adsorption, the number of hydroxyl groups in the structure of iron minerals decreased, with the hydroxyl groups in ferrihydrite decreasing the most, indicating that ferrihydrite has a strong affinity for PAA. The formation of a Fe-O-As bond is due to ligand exchange between the arsenate in PAA and the hydroxyl in the iron minerals, resulting in complexation.

## Figures and Tables

**Table 1:** Isothermal adsorption model fitting results of PAA adsorption by iron minerals.

Adsorbent	Langmuir			Freundlich			Redlich–Peterson			
	*Q*_max_ (mg/g)	*k*_L_	R^2^	*k*_F_	n	R^2^	*k*_R_	α	β	R^2^
Ferrihydrite	62.21	1.63	0.992	35.84	0.19	0.981	155	3.11	0.92	0.999
Goethite	22.99	0.13	0.996	6.21	0.314	0.982	2.14	0.036	1.24	0.999
Hematite	35.58	0.072	0.999	5.36	0.440	0.992	2.47	0.061	1.031	0.999

**Table 2:** Fitting results of the adsorption kinetics model of iron minerals.

Adsorbent	Pseudo First Order			Pseudo Second Order			Elovich		
	*Q*_e_/(mg/g)	*k*_1_	R^2^	*Q*_e_/(mg/g)	*k*_2_	R^2^	*α*	*β*	R^2^
Ferrihydrite	31.91	0.020	0.93	34.26	0.00085	0.98	3.87	0.19	0.99
Goethite	7.70	0.017	0.98	8.39	0.00267	0.99	0.48	0.70	0.95
Hematite	10.05	0.057	0.89	10.75	0.00725	0.96	6.78	0.75	0.99

**Table 3:** Fitting results of intragranular diffusion models of iron minerals.

Adsorbent	Stage I			Stage II			Stage III		
	*k*_1d_	*C*_1_	R^2^	*k*_2d_	*C*_2_	R^2^	*k*_3d_	*C*_3_	R^2^
Ferrihydrite	2.60	1.03	0.98	0.71	17.23	0.99	0.12	30.10	0.98
Goethite	0.70	−0.28	0.98	0.19	3.88	0.99	0.02	7.52	0.97
Hematite	1.26	0.57	0.93	0.14	7.23	0.99	0.08	8.70	0.96-

## Data Availability

Data will be made available on request.

## Supplementary Materials

The following supporting information can be downloaded at: https://www.mdpi.com/article/10.3390/molecules28083448/s1, Figure S1: The effect of humic acid on adsorption process of PAA with ferrihydrite; Figure S2: The ferrihydritereusability on adsorption process of PAA; Figure S3: PRB column experimental results; Figure S4: As 3d XPS spectra of ferrihydrite before and after adsorption of phenylarsonic acid; Figure S5: As 3d XPS spectra of hematite before and after adsorption of phenylarsonic acid; Figure S6: As 3d XPS spectra of goethite before and after adsorption of phenylarsonic acid; Figure S7: The adsorption mechanism diagram of PAA adsorption on iron minerals; Table S1: Fitting results of isothermal adsorption models of iron minerals at different temperatures; Table S2: The value of △G, △H, and △S obtained by Van’t Hoff curve.

## Sample Availability

Samples of ferrihydrite, goethite, and hematite are available from the authors.
