# Supplementary material for: Simple Co-Precipitation of Iron Minerals for the Removal of Phenylarsonic Acid: Insights into the Adsorption Performance and Mechanism
Source: Molecules. 2023 Apr 13;28(8):3448. doi: 10.3390/molecules28083448 (PMC10145160; doi:10.3390/molecules28083448)
Supplement: Supplementary file 1 [file molecules-28-03448-s001.zip › molecules-2299655-supplementary.pdf]

Supplementary Data

Simple co-precipitation of iron minerals for the removal of phenylarsonic acid: Insights into the adsorption performance and mechanism

Lili Wang[a], Changchao Hu[a,*], Ze Yang[b], Songding Guo[b], Tingting Zhang[b], Shangyi Li [b,*]

[a] Petroleum Exploration and Production Research Institute, SINOPEC, Beijing 100083, China; wangll.syky@sinopec.com(L.W.)

[b] Department of Environmental Science and Engineering, Research Centre for Resource and Environment, Beijing University of Chemical Technology, Beijing 100029, People's Republic of China; 1426149543@qq.com(Z.Y); guosongding123@163.com(S.G.); zhangtt@mail.buct.edu.cn(T.Z.)

*Correspondence: hucc2049@163.com (C.H.); lishangyi@mail.buct.edu.cn (S.L.); Tel.: +86-15810171131 (S.L.)

Contents:

Figure S1. The effect of humic acid on adsorption process of PAA with ferrihydrite.

Figure S2. The ferrihydritereusability on adsorption process of PAA.

Figure S3. PRB column experimental results

Figure S4. As 3d XPS spectra of ferrihydrite before and after adsorption of phenylarsonic acid.

Figure S5. As 3d XPS spectra of hematite before and after adsorption of phenylarsonic acid.

Figure S6. As 3d XPS spectra of goethite before and after adsorption of phenylarsonic acid.

Figure S7. The adsorption mechanism diagram of PAA adsorption on iron minerals.

Table S1. Fitting results of isothermal adsorption models of iron minerals at different temperatures.

Table S2. The value of △G, △H, and △S obtained by Van't Hoff curve.

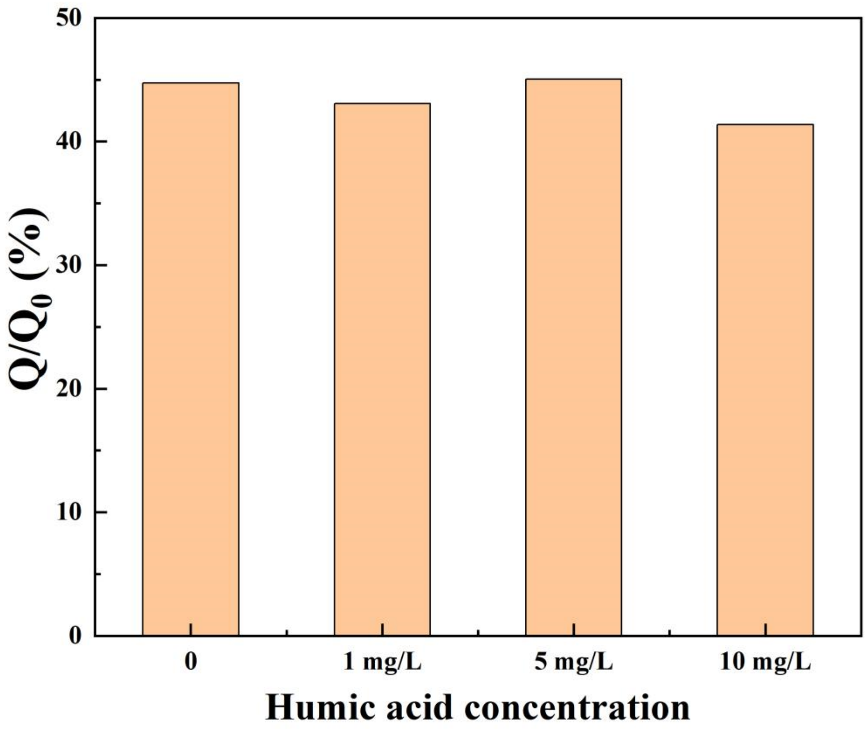

Figure S1. The effect of humic acid on adsorption process of PAA with ferrihydrite. (Q represented the adsorption capacity with humic acid in ferrihydrite/PAA and Q_0 represented the only ferrihydrite/PAA process)

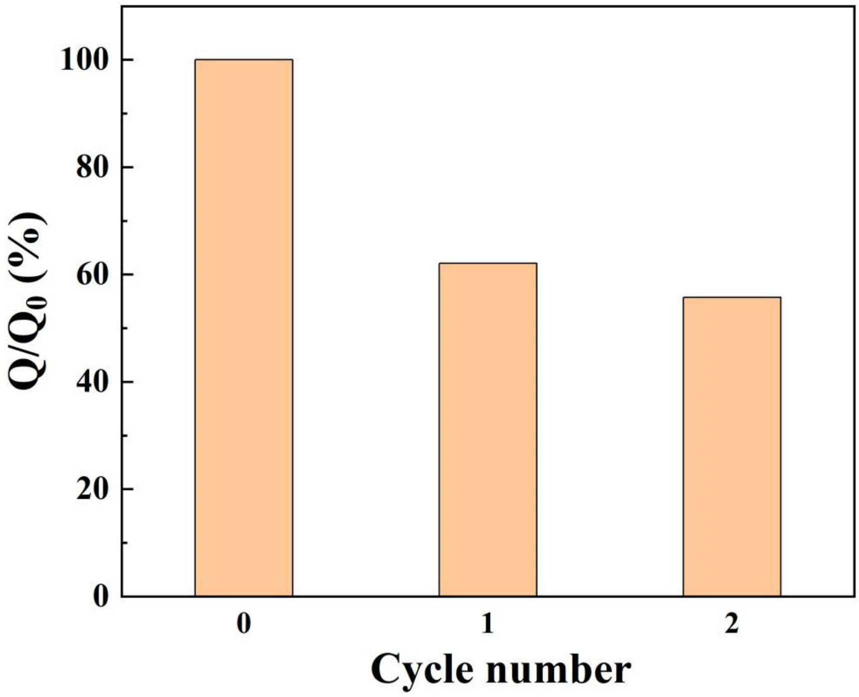

Figure S2. The ferrihydrite reusability on adsorption process of PAA. (Q represented the adsorption capacity by reused materials in ferrihydrite/PAA and Q_0 represented the initial ferrihydrite/PAA process)

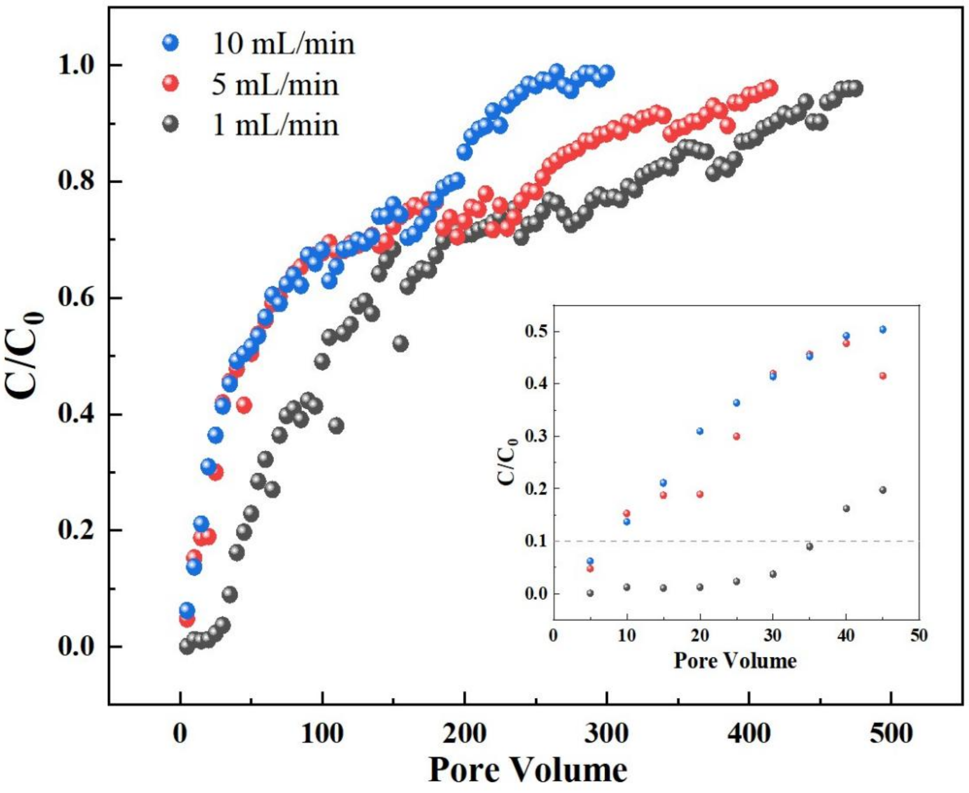

Figure S3. PRB column experimental results.

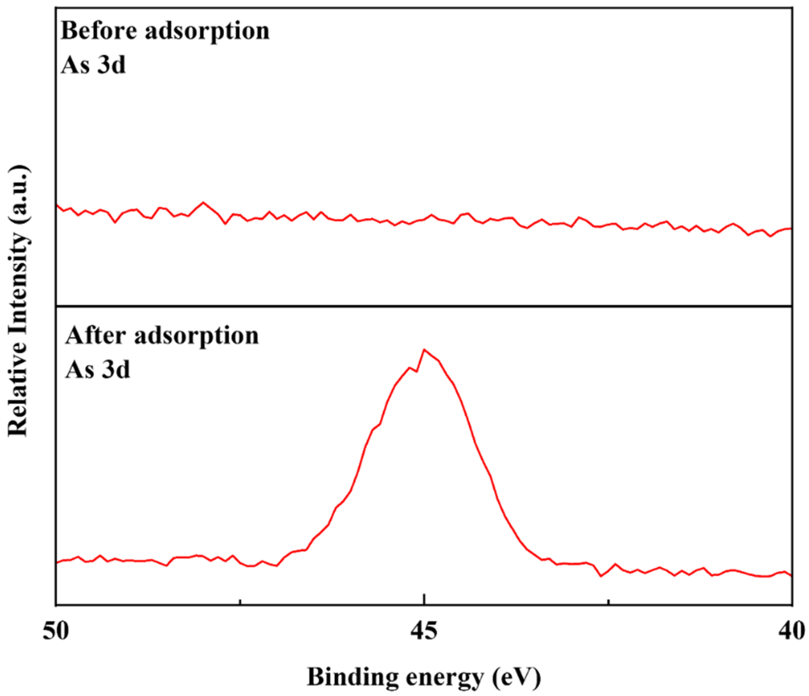

Figure S4. As 3d XPS spectra of ferrihydrite before and after adsorption of phenylarsonic acid.

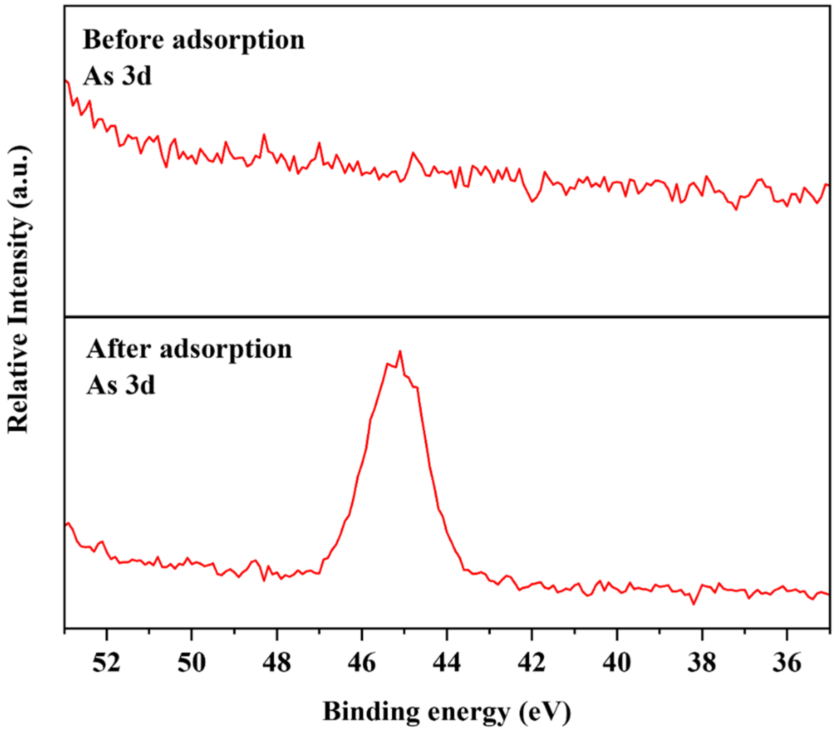

Figure S5. As 3d XPS spectra of hematite before and after adsorption of phenylarsonic acid.

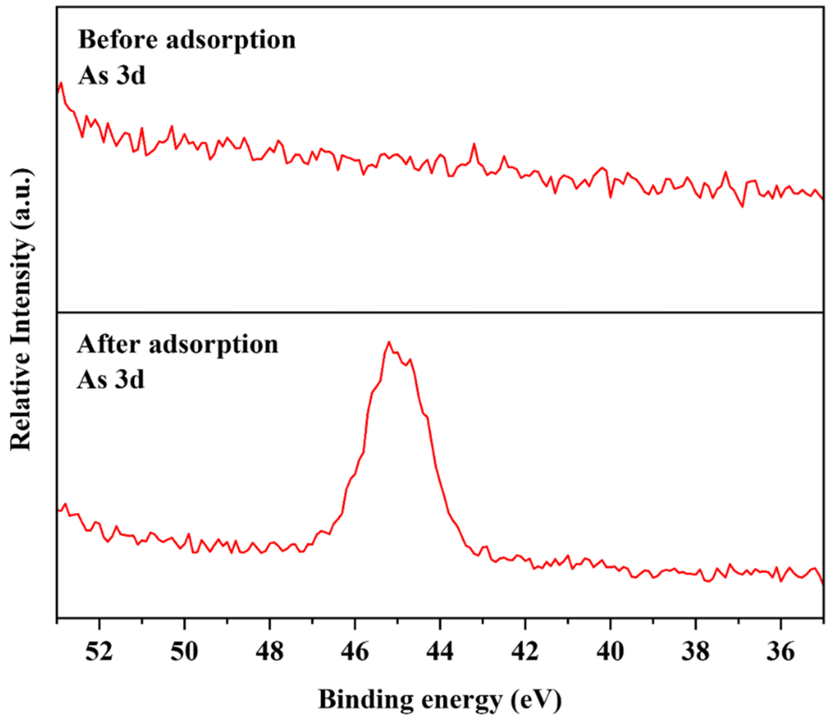

Figure S6. As 3d XPS spectra of goethite before and after adsorption of phenylarsonic acid.

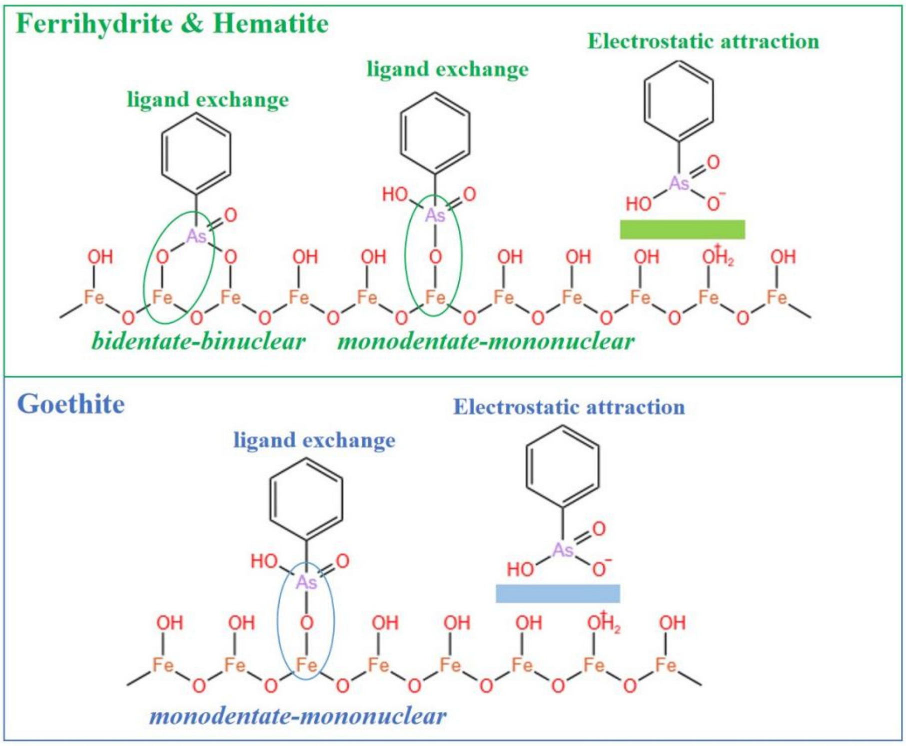

Figure S7. The adsorption mechanism diagram of PAA adsorption on iron minerals.

Table S1 Fitting results of isothermal adsorption models of iron minerals at different temperatures.

Adsorbent	Tempe-rature	Q_{max}	k_L	R^2	k_F	n	R^2	k_R	α	β	R^2
Ferrihydrite	308K	69.27	1.63	0.993	39.16	0.20	0.979	169.75	3.06	0.92	0.999
	298K	62.21	1.63	0.992	35.84	0.19	0.981	155	3.11	0.92	0.999
	288K	57.35	1.20	0.992	30.68	0.20	0.982	104	2.35	0.91	0.998
Goethite	308K	33.28	0.074	0.995	4.97	0.448	0.999	6.84	0.846	0.659	0.999
	298K	22.99	0.13	0.996	6.21	0.314	0.982	2.14	0.036	1.24	0.999
	288K	20.83	0.096	0.990	4.52	0.354	0.969	1.25	0.007	1.53	0.999
Hematite	308K	43.78	0.068	0.998	5.82	0.474	0.993	3.25	0.101	0.925	0.999
	298K	35.58	0.072	0.999	5.36	0.440	0.992	2.47	0.061	1.031	0.999
	288K	25.51	0.103	0.998	5.57	0.360	0.996	3.65	0.252	0.862	0.999

Table S2 The value of △G, △H, and △S obtained by Van't Hoff curve.

Materials	T（℃）	△G_0（kJ mol^{-1}）	△H_0（kJ mol^{-1}）	△S_0（J mol^{-1} K^{-1}）
	308	-20.67		
Ferrihydrite	298	-19.46	13.80	111.79
	288	-18.43		
	308	-16.62		
Goethite	298	-15.20	21.74	124.36
	288	-14.12		
	308	-17.25		
Hematite	298	-16.04	20.09	121.24
	288	-14.82		
